# Baseline Serum Uric Acid Levels Are Associated with All-Cause Mortality in Acute Coronary Syndrome Patients after Percutaneous Coronary Intervention

**DOI:** 10.1155/2018/9731374

**Published:** 2018-12-17

**Authors:** Ziliang Ye, Haili Lu, Manyun Long, Lang Li

**Affiliations:** ^1^Department of Cardiology, The First Affiliated Hospital of Guangxi Medical University, Nanning, Guangxi, China; ^2^Department of Orthodontic, The Affiliated Dental Hospital of Guangxi Medical University, Nanning, Guangxi, China

## Abstract

**Background:**

Whether serum uric acid (UA) is associated with all-cause mortality in patients with acute coronary syndrome (ACS) following percutaneous coronary intervention (PCI) remains unclear.

**Methods:**

We performed a retrospective cohort study of 2296 patients with ACS. Curve-fitting and Cox proportional-hazard regression models with a hazard ratio (HR) and 95% confidence interval (CI) were used.

**Results:**

During a mean follow-up of 246.31 ± 49.16 days, 168 (7.32%) patients died from all causes. Patients were divided into two groups [the high-UA group (*n* = 566) and the low-UA group (*n* = 1730)] based on the serum UA threshold value (5.6 mg/dl) identified through curve fitting. Fifty-three (9.36%) patients died in the high-UA group, and 115 (6.65%) patients died in the low-UA group. The difference between groups was statistically significant (*P* = 0.031). Univariate analysis showed that the risk of all-cause mortality in the high-UA group was significantly greater than that in the low-UA group (HR = 1.45, 95% CI: 1.03 to 2.04). This difference persisted after adjustment for baseline characteristics, medical history, and medication history (HR = 1.42, 95% CI: 1.05 to 1.87).

**Conclusions:**

Our study demonstrated that elevated serum UA (>5.6 mg/dl) is associated with all-cause mortality in ASC patients after PCI.

## 1. Introduction

Coronary heart disease (CHD) is caused by atherosclerosis accompanied by stenosis or lumen obstruction, resulting in myocardial ischemia, hypoxia, and necrosis [1–3]. As the standard of living continues to improve, the incidence of CHD is also increasing each year. CHD has become an important threat to human life and health [4]. Acute coronary syndrome (ACS) [5] is a serious type of CHD that can lead to adverse consequences, such as arrhythmia, heart failure, and sudden death [6–8]. ACS is divided into acute ST-elevation myocardial infarction (STEMI), non-ST-segment elevation myocardial infarction (NSTEMI), and unstable angina pectoris (UAP), depending on whether the patient has chest pain, the electrocardiogram findings, and the presence of markers of myocardial necrosis [9].

Previous studies have shown that coronary atherosclerotic plaque rupture, vasospasm and consequent platelet adhesion, aggregation, and secondary thrombosis are the main pathophysiological mechanisms of ACS [10, 11]. Additionally, diabetes [12], hypertension [13], obesity [14], smoking [15], and older age [16] are risk factors that promote the development and progression of ACS. A large number of studies have indicated that inflammatory factors are also closely related to ACS. For example, a cohort study in South Korea found that the level of plasma lipoprotein-associated phospholipase A2 (Lp-PLA2) and high-sensitivity C-reactive protein (hs-CRP) was significantly increased in patients with ACS. This relationship remained after a multivariate logistic regression analysis was performed to adjust for potential confounding factors (odds ratio = 1.047, *P* = 0.013) [17]. A systematic review that included 39,324 patients with ACS also found that baseline platelet (PLT) count was significantly associated with major adverse cardiac events (MACE) and mortality regardless of short-term (1-month follow-up) or long-term (>1 year) prognosis [18].

Uric acid (UA) is the final metabolite of nucleic acid, including nucleic acid from food, and excretion of UA occurs mainly through the kidneys [19, 20]. Any disorder of purine metabolism and/or abnormal excretion of UA can lead to an increase in blood UA. As medical research has advanced, it has been determined that UA is not only a metabolite of nucleic acid but also closely related to many diseases. Some scholars believe that higher levels of UA in patients with cardiovascular disease may be a compensatory response that counteracts excessive oxidative stress in the body [21, 22]. This theory is supported by animal research. Interestingly, a number of studies have shown that high UA levels are a risk factor for many diseases, such as hypertension [23] and cardiovascular diseases [24], and significantly worsen the prognosis of these patients, which might be explained by the close correlation between UA and oxidative stress [25]. However, it remains unclear whether serum UA is associated with all-cause mortality in ACS patients after percutaneous coronary intervention (PCI). Therefore, we performed this study to evaluate the association between serum UA and all-cause mortality in these patients.

## 2. Methods

### 2.1. Patients

This was a retrospective cohort study conducted at the First Affiliated Hospital of Guangxi Medical University, Guangxi, China, from February 2013 to January 2016. The study consecutively enrolled 2363 ACS patients who had undergone PCI. Sixty-seven patients were excluded from the study for the following reasons: 46 patients were excluded because left ventricular ejection fraction (LVEF) was not measured on admission or data was unavailable, 14 patients had a malignant tumor, 5 patients had severe liver and kidney disease, and 2 patients were excluded for other reasons. A total of 2296 patients were ultimately included in this study. This study was approved by the ethics committee of the First Affiliated Hospital of Guangxi Medical University, Guangxi, China. Because this was a retrospective cohort study and all patients were anonymous, written informed consent was not required. This study was conducted in accordance with the tenets of the Declaration of Helsinki. The data used to support the findings of this study are available from the corresponding author upon request.

The inclusion criteria were as follows: (1) the patient was diagnosed with ACS and underwent PCI from February 2013 to January 2016 at the First Affiliated Hospital of Guangxi Medical University, Guangxi, China, and (2) the patient's age was between 18 and 75 years. The exclusion criteria were as follows: (1) patients with malignant tumors, such as colorectal cancer, esophageal cancer, gastric cancer, or liver cancer; (2) renal function was severely impaired (estimated glomerular filtration rate < 30 ml/min/1.73 m^2^ or dialysis); (3) pregnant or nursing female patients; (4) patients with autoimmune diseases or blood disorders; or (5) data were not available for the hospitalization period after PCI.

### 2.2. Patient Information Collection

We used the electronic medical records of the First Affiliated Hospital of Guangxi Medical University to collect demographic characteristics, comorbidities, and cardiac medications of all patients. The patient information included age, sex, body mass index (BMI), systolic blood pressure (SBP), diastolic blood pressure (DBP), heart rate, serum creatinine, UA, total bilirubin, total cholesterol, triglycerides, high-density lipoprotein (HDL), low-density lipoprotein (LDL), LVEF, medical history, and medication history. Serum creatinine, UA, total bilirubin, total cholesterol, triglyceride, HDL, and LDL were measured within 24 hours after admission. Echocardiography was usually performed within 24 hours after admission by two echocardiography experts.

### 2.3. Treatment and Procedure

The drugs used before and after PCI were given in accordance with accepted guidelines and practice standards and included aspirin, clopidogrel, and statins. The PCI procedure and perioperative anticoagulant therapy were also carried out in accordance with accepted guidelines. The use of predilation, intravascular ultrasound, and intra-aortic balloon pumps, as well as the type of stent (drug-eluting or bare metal), was determined by experts in interventional cardiology.

### 2.4. Study Endpoints

The primary endpoint of this study was all-cause mortality, which was determined through the electronic medical records of the First Affiliated Hospital of Guangxi Medical University. If necessary, an office visit or telephone call was placed to confirm the clinical outcome of patients. All clinical outcomes were confirmed by two clinicians.

### 2.5. Statistical Analysis

**Figure 1:**
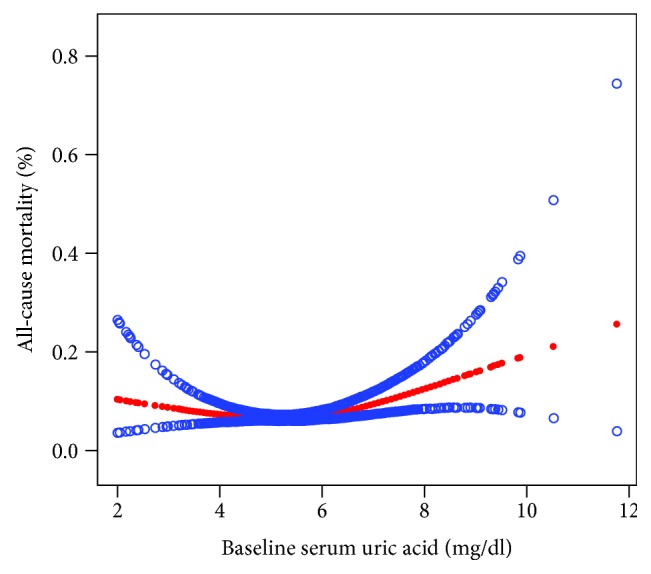
Curve fitting of baseline serum uric acid levels and all-cause mortality in ACS patients after PCI. Curve fitting found that when serum uric acid levels are below 5.6 mg/dl, the all-cause mortality rate is the lowest.

**Figure 2:**
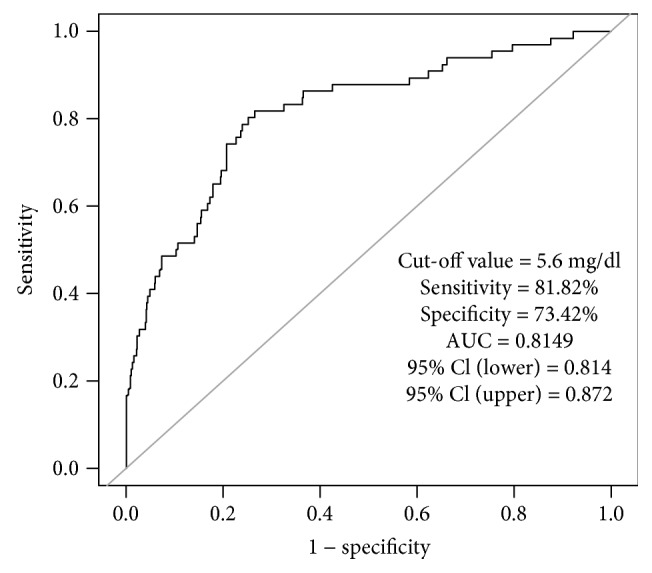
Receiver operating characteristic curve. The result of ROC shows that the cut-off value of serum uric acid for predicting all-cause mortality is 5.6 mg/dl [sensitivity = 81.82%, specificity = 73.42%, area under curve (AUC) = 81.52%, 95% CI (lower) = 0.814, and 95% CI (upper) = 0.872].

Results are presented as a number (percent) for categorical variables and as the mean ± SD for continuous variables. Categorical variables were compared using the chi-square test, and between-group comparisons of continuous variables were done using the unpaired Student's *t*-test. In this study, we used a curve-fitting method to explore the relationship between serum UA and all-cause mortality. We found the association between serum UA and all-cause mortality to be U-shaped. This means that all-cause mortality decreased first and then increased as serum UA increased, with a threshold value of 5.6 mg/dl (Figure 1). In addition, the receiver operating characteristic (ROC) curve was performed to further validate the relationship between the uric acid level and all-cause mortality. The result of ROC also shows that the cut-off value of serum uric acid for predicting all-cause mortality is 5.6 mg/dl [sensitivity = 81.82%, specificity = 73.42%, area under curve (AUC) = 81.52%, 95% CI (lower) = 0.814, and 95% CI (upper) = 0.872] (Figure 2). Finally, all patients were divided into two groups [the high-UA group (*n* = 566) and the low-UA group (*n* = 1730)] depending on their level of serum UA (threshold value = 5.6 mg/dl) through curve fitting and ROC.

We also performed univariate and multivariate logistic regression analyses to estimate all-cause mortality between the two groups. In multivariate logistic regression analysis, demographic and clinical factors, including age, sex, BMI, SBP, DBP, heart rate, serum creatinine, total bilirubin, total cholesterol, triglyceride, HDL, LDL, LVEF, medical history, and medication history, were adjusted to obtain accurate results. Additionally, a Cox proportional-hazard regression model was used to explore the association between baseline serum uric acid levels and all-cause mortality. A hazard ratio (HR) with a 95% confidence interval (CI) was used to estimate the results.

Considering the differences in serum UA levels between sex and different diseases, we further conducted a subgroup analysis to explore the relationship between serum UA levels and all-cause mortality based on sex, history of heart failure, history of atrial fibrillation, history of stroke, history of PCI, history of hypertension, history of diabetes, and smoking.

All reported probability values were 2-sided with a *P* value < 0.05 considered to be statistically significant. Data analysis was performed using IBM SPSS Statistics for Windows, version 21.0 (IBM Corp., Armonk, NY, USA), R statistical software version 3.3.2 (available at: http://www.r-project.org), and EmpowerStats (http://www.empowerstats.com).

## 3. Results

### 3.1. Baseline Characteristics of Study Patients

From February 2013 to January 2016, 2296 ACS patients who had undergone PCI were consecutively enrolled in the study. During a mean follow-up of 246.31 ± 49.16 days, 168 (7.32%) patients died from all causes. Through curve fitting and the result of ROC, all patients were divided into two groups [the high-UA group (*n* = 566) and the low-UA group (*n* = 1730)] according to the level of serum UA (threshold value = 5.6 mg/dl) (Figures 1 and 2). Baseline characteristics of the two groups are presented in Table 1. There were no significant differences in age, BMI, SBP, DBP, heart rate, total bilirubin, total cholesterol, HDL, LDL, LVEF, history of heart failure, history of atrial fibrillation, history of stroke, history of PCI, history of CABG, history of hypertension, smoking, or use of aspirin, clopidogrel, beta-blockers, ACEIs, calcium channel blockers, or statins between the low-UA group and the high-UA group (all *P* > 0.05). However, the high-UA group had higher serum creatinine and triglycerides and a lower prevalence of diabetes compared with the low-UA group (*P* < 0.05). In addition, the proportion of females in the low-UA group was higher than that in the high-UA group (35.84% vs. 20.85%).

### 3.2. Follow-Up and All-Cause Mortality

During a mean follow-up of 246.31 ± 49.16 days, 168 (7.32%) patients died from all causes. Fifty-three (9.36%) of these patients were in the high-UA group, and 115 (6.65%) were in the low-UA group. The difference in all-cause mortality rates was statistically significant between the groups (*P* = 0.031).

### 3.3. Univariate and Multivariate Analyses

In univariate analyses, we found sex (HR = 0.68, 95% CI: 0.49 to 0.93), age (HR = 1.09, 95% CI: 1.07 to 1.11), history of heart failure (HR = 3.12, 95% CI: 2.16 to 4.50), history of atrial fibrillation (HR = 2.68, 95% CI: 1.23 to 5.83), history of diabetes (HR = 2.13, 95% CI: 1.53 to 2.98), serum creatinine (HR = 1.06, 95% CI: 1.02 to 1.21), and baseline serum UA (HR = 1.45, 95% CI: 1.03 to 2.04) to be significantly associated with all-cause mortality (Table 2). When potential confounding factors were adjusted for through multivariate analysis, sex (HR = 0.73, 95% CI: 0.54 to 0.85), age (HR = 1.11, 95% CI: 1.09 to 1.14), history of heart failure (HR = 3.10, 95% CI: 2.15 to 4.47), history of atrial fibrillation (HR = 2.65, 95% CI: 1.22 to 5.78), history of diabetes (HR = 2.17, 95% CI: 1.55 to 3.04), and baseline serum uric acid (HR = 1.42, 95% CI: 1.05 to 1.87) were still significantly associated with all-cause mortality (Table 2).

### 3.4. Cox Proportional-Hazard Regression Model

**Figure 3:**
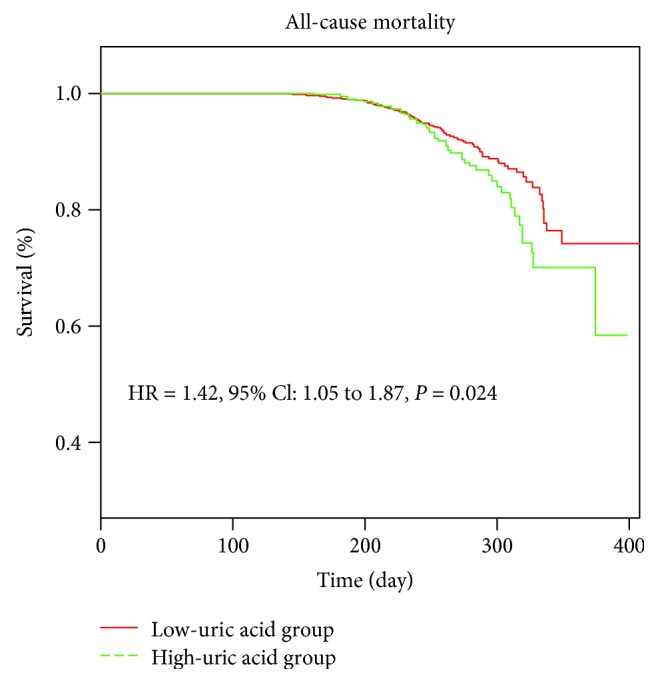
Cox proportional-hazard regression model (HR = 1.42, 95% CI: 1.05 to 1.87) for all-cause mortality based on baseline serum uric acid levels during a mean follow-up period of 246.31 ± 49.16 days, adjusted for serum creatinine, triglyceride, and history of heart failure.

For a mean follow-up of 246.31 ± 49.16 days, the results using the Cox regression model are shown in Figure 3. Compared with patients in the low-uric acid group (uric acid < 5.6 mg/dl), patients in the high-uric acid group (uric acid > 5.6 mg/dl) had a 42% increased risk of all-cause mortality after adjustment for baseline characteristics, medical history, and medication history (HR = 1.42, 95% CI: 1.05 to 1.87).

### 3.5. Subgroup Analysis

In subgroup analysis, we found that serum UA levels are associated with all-cause mortality in ACS patients after PCI, regardless of male (HR: 0.71, 95% CI: 0.62 to 0.87) or female (HR: 0.77, 95% CI: 0.54 to 0.92). Besides, subgroup analysis also illuminated that patients with a history of hypertension heart failure (HR: 2.32, 95% CI: 1.22 to 4.42), atrial fibrillation (HR: 1.63, 95% CI: 1.13 to 3.01), and diabetes (HR: 1.67, 95% CI: 1.16 to 2.89) will have a higher risk of all-cause mortality (Table 3).

## 4. Discussion

Patients with ACS often suffer from an increased risk of all-cause mortality and reduced survival time after PCI. Previous studies have found that the risk of all-cause mortality in patients with ACS was 20.34% within one year after PCI [26]. Therefore, one of the key concerns of the clinician is to identify and manage risk factors in order to minimize the risk of mortality. To date, researchers have found that vitamin D levels [27], education level [28], posttraumatic stress [29], and adiponectin [30] are risk predictors of all-cause mortality.

In this retrospective cohort study, our data reveal that patients with a higher level of UA at baseline (threshold value > 5.6 mg/dl) had a higher risk of all-cause mortality compared to patients with a lower level of UA. Similar results were attained when potentially confounding factors (such as baseline characteristics, medical history, and medication history) were adjusted using multivariate logistic regression analysis and Cox proportional-hazard regression model (HR = 1.42, 95% CI: 1.05 to 1.87), which means that the UA level might be used to better stratify cardiovascular risk in clinical practice. To the best of our knowledge, this retrospective cohort study of 2296 patients is the first to evaluate the association between baseline serum UA levels and all-cause mortality in a Chinese population of ACS patients after PCI.

Our results suggest that as the UA level increases, the incidence of all-cause mortality also increases. This means that there is a positive correlation between the level of UA and all-cause mortality in ACS patients after PCI. This finding is very important for patients, the social health care system, and the National Health and Family Planning Commission and warrants more attention and support. Early intervention and management in order to reduce risk is important to improve the prognosis of these patients in the long term.

In recent years, it has gradually been found that UA is a risk factor for many diseases, such as dementia [31], hypertension [32], diabetes [33], and coronary heart disease [34]. Perticone et al. found that UA is an independent predictor of cardiovascular outcomes and increases prognostic accuracy of Cox models in patients with essential hypertension [35]. A retrospective study pointed out that the incidence of CVD was increased along with an increase in UA levels in both men and women. The hazard ratio for CVD was 1.24 (95% confidence interval 1.08 to 1.41) for women and 1.06 (95% confidence interval 1.00 to 1.13) for men [36]. Another population-based cross-sectional study observed that elevated serum UA levels were associated with CVD independent of traditional cardiovascular risk factors (*P* < 0.05) [37]. Consistent with previous studies, our study found that UA levels are a risk factor for all-cause mortality, independent of other cardiovascular risk factors.

The exact mechanisms to explain the relationship between uric acid and all-cause mortality are as follows. Some scholars suggest that high UA levels promote the occurrence and development of atherosclerosis [38–40]. It is well known that atherosclerosis is closely related to the incidence of cardiovascular disease and therefore could lead to an increased risk of all-cause mortality. Other scholars suggest that elevated serum UA may lead to endothelial dysfunction [41, 42]. Blood UA can interfere with the synthesis of nitric oxide, inhibit nitric oxide bioavailability, activate the renin-angiotensin system, promote vascular smooth muscle cell proliferation and platelet aggregation, and eventually lead to the endothelial dysfunction [43, 44]. Additionally, some previous studies have found that there is a large amount of UA in atherosclerotic plaque, which can increase the adhesion of platelets and promote the formation of thrombus, thereby affecting the patient prognosis and increasing all-cause mortality [45].

Previous studies have showed that elevated uric acid levels appear to significantly increase the risk of all-cause mortality in men, but not in women, which is inconsistent with our results [46]. The possible explanations are as follows: (1) different diseases may lead to inconsistent results. The subjects of this study were acute coronary syndrome patients following percutaneous coronary intervention. However, the subjects of some previous research were patients treated with peritoneal dialysis. (2) The difference in research population is also one of the reasons leading to inconsistent results. The object of this study is the Chinese population; perhaps because of different genes or eating habits, it can lead to different results.

Our study has several strengths. First, to the best of our knowledge, this is the first study to evaluate the association between baseline serum UA and all-cause mortality in ACS patients after PCI. The implication for clinical practice is that taking appropriate measures to reduce UA levels may significantly reduce all-cause mortality in these patients. Serum UA levels are part of the routine examinations of inpatients and can easily be addressed using sodium bicarbonate, which is low cost. Second, the threshold value for serum UA levels (5.6 mg/dl) was determined through curve fitting, which better represents the real-world situation compared to the traditional grouping method. Finally, although this was a retrospective cohort study, we performed a multivariate logistic regression analysis to minimize residual confounding, including adjustment for demographic and clinical factors.

Our study also has several limitations. First, as a retrospective cohort study, there is inevitably some confounding bias, such as selection bias and retrospect bias. Second, this research is a single-center study in a Chinese population. Further studies are needed to confirm whether these results are applicable in other areas and, especially, other populations. Finally, some previous baseline data were not available. For example, data regarding the degree of coronary artery disease stenosis, type of stent implantation, and the number of implanted stents were not available.

## 5. Conclusion

Our study demonstrated that elevated serum UA (>5.6 mg/dl) is associated with all-cause mortality in ASC patients after PCI. However, more studies are needed to confirm this finding.

## Figures and Tables

**Table 1:** Baseline characteristics of study patients.

Variables	Low uric acid group	High uric acid group	*P* value
*N*	1730	566	
Age (year)	60.05 ± 10.72	60.22 ± 12.09	0.744
BMI (kg/m^2^)	23.86 ± 3.98	23.86 ± 3.69	0.986
SBP (mmHg)	102.84 ± 28.67	104.28 ± 28.98	0.302
DBP (mmHg)	77.87 ± 11.93	78.38 ± 12.41	0.382
Heart rate (times/min)	71.87 ± 11.70	73.14 ± 11.68	0.030
Serum creatinine (*μ*mol/l)	68.81 ± 31.86	83.97 ± 37.94	<0.001
Total bilirubin (*μ*mol/l)	9.67 ± 7.76	10.45 ± 6.27	0.030
Total cholesterol (mmol/l)	4.25 ± 1.05	4.29 ± 1.09	0.436
Triglyceride (mmol/l)	1.82 ± 1.24	2.20 ± 1.63	<0.001
HDL (mmol/l)	1.08 ± 0.32	1.06 ± 0.29	0.186
LDL (mmol/l)	2.67 ± 0.93	2.68 ± 0.93	0.780
LVEF (%)	60.98 ± 7.32	60.57 ± 7.66	0.361
Sex, female (%)	620 (35.84%)	118 (20.85%)	<0.001
History of heart failure, yes (%)	202 (11.70%)	73 (12.90%)	0.448
History of atrial fibrillation, yes (%)	37 (2.14%)	10 (1.77%)	0.588
History of stroke, yes (%)	94 (5.43%)	29 (5.12%)	0.776
History of PCI, yes (%)	116 (6.71%)	33 (5.84%)	0.469
History of hypertension, yes (%)	862 (49.83%)	292 (51.59%)	0.348
History of diabetes, yes (%)	398 (23.03%)	101 (17.84%)	0.009
Smoking	537 (31.04%)	187 (33.04%)	0.531
Aspirin, yes (%)	1706 (98.73%)	560 (98.94%)	0.689
Clopidogrel, yes (%)	1658 (95.89%)	541 (95.58%)	0.949
Beta-blockers, yes (%)	1218 (70.40%)	393 (69.43%)	0.662
ACEI, yes (%)	939 (54.28%)	325 (57.52%)	0.178
Calcium channel blocker, yes (%)	423 (24.45%)	143 (25.27%)	0.696
Statin, yes (%)	1627 (94.05%)	525 (92.76%)	0.272

Abbreviations: BMI = body mass index; SBP = systolic blood pressure; DBP = diastolic blood pressure; HDL = high-density lipoprotein; LDL = low-density lipoprotein; LVEF = left ventricular ejection fraction; PCI = percutaneous coronary intervention; CABG = cardiac artery bypass grafting; ACEI = angiotensin-converting enzyme inhibitor.

**Table 2:** Univariate and multivariate analyses of the relationship between uric acid and all-cause mortality.

	Univariate analysis			Multivariate analyses		
	HR	95% CI	*P* value	HR	95% CI	*P* value
Sex	0.68	(0.49, 0.93)	0.017	0.73	(0.54, 0.85)	0.012
Age	1.09	(1.07, 1.11)	<0.001	1.11	(1.09, 1.14)	<0.001
History of heart failure	3.12	(2.16, 4.50)	<0.001	3.10	(2.15, 4.47)	<0.001
History of atrial fibrillation	2.68	(1.23, 5.83)	0.013	2.65	(1.22, 5.78)	0.014
History of stroke	1.40	(0.75, 2.59)	0.287	—	—	—
History of PCI	0.70	(0.34, 1.46)	0.346	—	—	—
History of hypertension	1.04	(0.76, 1.42)	0.807	—	—	—
History of diabetes	2.13	(1.53, 2.98)	<0.001	2.17	(1.55, 3.04)	<0.001
Smoking	0.97	(0.69, 1.35)	0.841	—	—	—
BMI	0.92	(0.88, 1.09)	0.215	—	—	—
SBP	1.03	(0.92, 1.06)	0.085	—	—	—
DBP	1.07	(0.94, 1.12)	0.613	—	—	—
Heart rate	0.88	(0.99, 1.22)	0.851	—	—	—
Serum creatinine	1.06	(1.02, 1.21)	0.005	0.87	(0.76, 1.13)	0.263
Total bilirubin	0.94	(1.00, 1.03)	0.123	—	—	—
Total cholesterol	0.89	(0.76, 1.04)	0.137	—	—	—
Triglyceride	0.81	(0.68, 0.96)	0.013	0.84	(0.76, 1.23)	0.071
HDL	0.78	(0.46, 1.34)	0.370	—	—	—
LDL	1.01	(0.85, 1.20)	0.901	—	—	—
LVEF	0.91	(0.84, 1.26)	0.342	—	—	—
Baseline serum uric acid	1.45	(1.03, 2.04)	0.032	1.42	(1.05, 1.87)	0.024

Abbreviations: BMI = body mass index; SBP = systolic blood pressure; DBP = diastolic blood pressure; HDL = high-density lipoprotein; LDL = low-density lipoprotein; LVEF = left ventricular ejection fraction; PCI = percutaneous coronary intervention; CABG = cardiac artery bypass grafting; ACEI = angiotensin-converting enzyme inhibitor. Multivariate analyses: adjusted for serum creatinine, triglyceride, and history of heart failure.

**Table 3:** Subgroup analysis.

Subgroup	All-cause mortality		
	HR	95% CI	*P* value
Sex			
Male	0.71	(0.62, 0.87)	0.016
Female	0.77	(0.54, 0.92)	0.022
Heart failure			
No	0.91	(0.62, 1.33)	0.626
Yes	2.32	(1.22, 4.42)	0.010
Atrial fibrillation			
No	1.19	(0.86, 1.65)	0.288
Yes	1.63	(1.13, 3.01)	0.027
Stroke			
No	1.20	(0.86, 1.67)	0.276
Yes	0.85	(0.24, 3.01)	0.805
History of PCI			
No	1.20	(0.87, 1.66)	0.272
Yes	0.79	(0.18, 3.42)	0.749
Hypertension			
No	1.14	(0.72, 1.80)	0.584
Yes	1.20	(0.77, 1.86)	0.422
Diabetes			
No	1.06	(0.72, 1.58)	0.756
Yes	1.67	(1.16, 2.89)	0.027
Smoking			
No	1.32	(0.89, 1.94)	0.163
Yes	0.93	(0.53, 1.62)	0.793

PCI = percutaneous coronary intervention.

## Data Availability

The data used to support the findings of this study are available from the corresponding author upon request.

## References

[1] Unal B., Sözmen K., Arık H. (2013). Explaining the decline in coronary heart disease mortality in Turkey between 1995 and 2008. *BMC Public Health*.

[2] Cannon C. P., Shah S., Dansky H. M. (2010). Safety of anacetrapib in patients with or at high risk for coronary heart disease. *The New England Journal of Medicine*.

[3] Polonsky T. S., Mcclelland R. L., Jorgensen N. W., Bild D. E., Burke G. L., Guerci A. D. (2010). Coronary artery calcium score and risk classification for coronary heart disease prediction. *JAMA*.

[4] Wong N. D. (2014). Epidemiological studies of CHD and the evolution of preventive cardiology. *Nature Reviews Cardiology*.

[5] Mega J. L., Braunwald E., Wiviott S. D. (2012). Rivaroxaban in patients with a recent acute coronary syndrome. *The New England Journal of Medicine*.

[6] Alfaleh H. F., Alhabib K. F., Kashour T. (2014). Short-term and long-term adverse cardiovascular events across the glycaemic spectrum in patients with acute coronary syndrome: the Gulf Registry of Acute Coronary Events-2. *Coronary Artery Disease*.

[7] Chen Y. H., Liu J. M., Hsu R. J. (2012). Angiotensin converting enzyme DD genotype is associated with acute coronary syndrome severity and sudden cardiac death in Taiwan: a case-control emergency room study. *Bmc Cardiovascular Disorders.*.

[8] Ye Z., Lu H., Su Q. (2017). Effect of high-dose rosuvastatin loading before percutaneous coronary intervention in Chinese patients with acute coronary syndrome: a systematic review and meta-analysis. *PLoS One*.

[9] Cecchi E., Liotta A. A., Gori A. M. (2008). Comparison of hemorheological variables in ST-elevation myocardial infarction versus those in non-ST-elevation myocardial infarction or unstable angina pectoris. *The American Journal of Cardiology*.

[10] Gonzi G., Merlini P. A., Ardissino D. (2001). Invasive coronary revascularisation is better than conservative treatment in patients with acute coronary syndromes. *Heart*.

[11] Sakaguchi M., Hasegawa T., Ehara S. (2016). New insights into spotty calcification and plaque rupture in acute coronary syndrome: an optical coherence tomography study. *Heart and Vessels*.

[12] Dong X., Cai R., Sun J. (2017). Diabetes as a risk factor for acute coronary syndrome in women compared with men: a meta-analysis, including 10, 856, 279 individuals and 106, 703 acute coronary syndrome events. *Diabetes/metabolism Research and Reviews*.

[13] Picariello C., Lazzeri C., Attanà P., Chiostri M., Gensini G. F., Valente S. (2011). The impact of hypertension on patients with acute coronary syndromes. *International Journal of Hypertension*.

[14] Diercks D. B., Roe M. T., Mulgund J. (2006). The obesity paradox in non–ST-segment elevation acute coronary syndromes: results from the Can Rapid Risk Stratification of Unstable Angina Patients Suppress Adverse Outcomes with Early Implementation of the American College of Cardiology/American Heart Association Guidelines Quality Improvement Initiative. *American Heart Journal*.

[15] Yagi H., Komukai K., Hashimoto K. (2010). Difference in risk factors between acute coronary syndrome and stable angina pectoris in the Japanese: smoking as a crucial risk factor of acute coronary syndrome. *Journal of Cardiology*.

[16] Zaman M. J., Stirling S., Shepstone L. (2014). The association between older age and receipt of care and outcomes in patients with acute coronary syndromes: a cohort study of the Myocardial Ischaemia National Audit Project (MINAP). *European Heart Journal*.

[17] Hyemoon C., Moon K. H., Jong-Youn K., Won Y. Y., Jihyuk R., Eui-Young C. (2014). Lipoprotein-associated phospholipase A2Is related to plaque stability and is a potential biomarker for acute coronary syndrome. *Yonsei Medical Journal*.

[18] Wu Y., Wu H., Mueller C. (2012). Baseline platelet count and clinical outcome in acute coronary syndrome. *Circulation Journal*.

[19] Nagura M., Tamura Y., Kumagai T., Hosoyamada M., Uchida S. (2016). Uric acid metabolism of kidney and intestine in a rat model of chronic kidney disease. *Nucleosides & Nucleotides*.

[20] Verdoia M., Barbieri L., Schaffer A. (2014). Impact of diabetes on uric acid and its relationship with the extent of coronary artery disease and platelet aggregation: a single-centre cohort study. *Metabolism Clinical and Experimental*.

[21] Chiquete E., Ruiz-Sandoval J. L., Murillo-Bonilla L. M. (2013). Serum uric acid and outcome after acute ischemic stroke: PREMIER study. *Cerebrovascular Diseases*.

[22] Miedema I., Uyttenboogaart M., Koch M., Kremer B., de Keyser J., Luijckx G-J. (2012). Lack of association between serum uric acid levels and outcome in acute ischemic stroke. *Journal of the Neurological Sciences*.

[23] Yokokawa H., Fukuda H., Suzuki A. (2016). Association between serum uric acid levels/hyperuricemia and hypertension among 85, 286 Japanese workers. *Journal of Clinical Hypertension*.

[24] Lotufo P. A., Baena C. P., Santos I. S., Bensenor I. M. (2016). Serum uric acid and prehypertension among adults free of cardiovascular diseases and diabetes: baseline of the Brazilian Longitudinal Study of Adult Health (ELSA-Brasil). *Angiology*.

[25] Lanaspa M. A., Sanchezlozada L. G., Choi Y. J. (2012). Uric acid induces hepatic steatosis by generation of mitochondrial oxidative stress: potential role in fructose-dependent and -independent fatty liver. *Journal of Biological Chemistry*.

[26] Kilcullen N., Viswanathan K., Das R. (2007). Heart-type fatty acid-binding protein predicts long-term mortality after acute coronary syndrome and identifies high-risk patients across the range of troponin values. *Journal of the American College of Cardiology*.

[27] Naesgaard P. A., León de la Fuente R. A., Nilsen S. T. (2013). Vitamin d predicts all-cause and cardiac mortality in females with suspected acute coronary syndrome: a comparison with brain natriuretic peptide and high-sensitivity C-reactive protein. *Cardiology research and practice*.

[28] Abbasi S., De Leon A., Kassaian S. (2015). Socioeconomic status and in-hospital mortality of acute coronary syndrome: can education and occupation serve as preventive measures. *International Journal of Preventive Medicine*.

[29] Edmondson D., Rieckmann N., Shaffer J. A. (2011). Posttraumatic stress due to an acute coronary syndrome increases risk of 42-month major adverse cardiac events and all-cause mortality. *Journal of Psychiatric Research*.

[30] Nakashima H., Henmi T., Minami K. (2013). Adiponectin is the most useful predictor for all-cause mortality and recurrence of acute coronary syndrome in patients with acute myocardial infarction. *European Heart Journal*.

[31] Euser S. M., Hofman A., Westendorp R. G. J., Breteler M. M. B. (2009). Serum uric acid and cognitive function and dementia. *Brain*.

[32] Johnson R. J., Sánchez-Lozada L. G., Mazzali M., Feig D. I., Kanbay M., Sautin Y. Y. (2013). What are the key arguments against uric acid as a true risk factor for hypertension?. *Hypertension*.

[33] Dehghan A., Hoek M. V., Sijbrands E. J. G., Hofman A., Witteman J. C. M. (2008). High serum uric acid as a novel risk factor for type 2 diabetes. *Diabetes Care*.

[34] Barbieri L., Verdoia M., Schaffer A., Marino P., Suryapranata H., Luca G. D. (2015). Impact of sex on uric acid levels and Its relationship with the extent of coronary artery disease: a single-centre study. *Atherosclerosis*.

[35] Perticone M., Tripepi G., Maio R. (2017). Risk reclassification ability of uric acid for cardiovascular outcomes in essential hypertension. *International Journal of Cardiology*.

[36] Kivity S., Kopel E., Maor E. (2013). Association of serum uric acid and cardiovascular disease in healthy adults. *The American Journal of Cardiology*.

[37] Qin L., Yang Z., Gu H. (2014). Association between serum uric acid levels and cardiovascular disease in middle-aged and elderly Chinese individuals. *Bmc Cardiovascular Disorders.*.

[38] Johnson R. J., Rodriguez-Iturbe B., Kang D. H., Feig D. I., Herrera-Acosta J. (2005). A unifying pathway for essential hypertension. *American Journal of Hypertension*.

[39] Ishizaka N., Ishizaka Y., Toda E. I., Nagai R., Yamakado M. (2005). Association between serum uric acid, metabolic syndrome, and carotid atherosclerosis in Japanese individuals. *Arteriosclerosis, Thrombosis, and Vascular Biology*.

[40] Kawamoto R., Tomita H., Oka Y., Ohtsuka N. (2006). Relationship between serum uric acid concentration, metabolic syndrome and carotid atherosclerosis. *Internal Medicine*.

[41] Kanbay M., Yilmaz M. I., Sonmez A. (2011). Serum uric acid level and endothelial dysfunction in patients with nondiabetic chronic kidney disease. *American Journal of Nephrology*.

[42] Perticone F., Maio R., Tassone J. E. (2013). Interaction between uric acid and endothelial dysfunction predicts new onset of diabetes in hypertensive patients. *International Journal of Cardiology*.

[43] Kanellis J., Kang D. H. (2005). Uric acid as a mediator of endothelial dysfunction, inflammation, and vascular disease. *Seminars in Nephrology*.

[44] Yu M. A., Sánchezlozada L. G., Johnson R. J., Kang D. H. (2010). Oxidative stress with an activation of the renin–angiotensin system in human vascular endothelial cells as a novel mechanism of uric acid-induced endothelial dysfunction. *Journal of Hypertension*.

[45] Mercuro G., Vitale C., Cerquetani E. (2004). Effect of hyperuricemia upon endothelial function in patients at increased cardiovascular risk. *The American Journal of Cardiology*.

[46] Zhao G., Huang L., Song M., Song Y. (2013). Baseline serum uric acid level as a predictor of cardiovascular disease related mortality and all-cause mortality: a meta-analysis of prospective studies. *Atherosclerosis*.

